# The Role of Neutrophils and Neutrophil Elastase in Pulmonary Arterial Hypertension

**DOI:** 10.3389/fmed.2018.00217

**Published:** 2018-08-03

**Authors:** Shalina Taylor, Omar Dirir, Roham T. Zamanian, Marlene Rabinovitch, A. A. Roger Thompson

**Affiliations:** ^1^Department of Pediatrics, Stanford University School of Medicine, Stanford, CA, United States; ^2^Vera Moulton Wall Center for Pulmonary Vascular Disease, Stanford University, Stanford, CA, United States; ^3^Infection, Immunity, and Cardiovascular Disease, University of Sheffield, Sheffield, United Kingdom; ^4^Division of Pulmonary and Critical Care Medicine, Stanford University School of Medicine, Stanford, CA, United States

**Keywords:** neutrophils, neutrophil elastase, pulmonary hypertension, pulmonary arterial hypertension, vascular remodeling

## Abstract

Pulmonary arterial hypertension (PAH) is a severe vasculopathy characterized by the presence of fibrotic lesions in the arterial wall and the loss of small distal pulmonary arteries. The vasculopathy is accompanied by perivascular inflammation and increased protease levels, with neutrophil elastase notably implicated in aberrant vascular remodeling. However, the source of elevated elastase levels in PAH remains unclear. A major source of neutrophil elastase is the neutrophil, an understudied cell population in PAH. The principal function of neutrophils is to destroy invading pathogens by means of phagocytosis and NET formation, but proteases, chemokines, and cytokines implicated in PAH can be released by and/or prime and activate neutrophils. This review focuses on the contribution of inflammation to the development and progression of the disease, highlighting studies implicating neutrophils, neutrophil elastase, and other neutrophil proteases in PAH. The roles of cytokines, chemokines, and neutrophil elastase in the disease are discussed and we describe new insight into the role neutrophils potentially play in the pathogenesis of PAH.

## Pulmonary arterial hypertension and inflammation

Pulmonary arterial hypertension (PAH) is a progressive disease characterized by thickening, and progressive occlusion of distal arteries in the lung related to vascular cell dysfunction, and perivascular inflammation. As a consequence of elevation in pulmonary arterial pressure, the right ventricle hypertrophies and later becomes dysfunctional leading to right heart failure (1, 2). Perivascular inflammation has been observed in all subsets of PAH and correlates with clinical markers of disease progression such as an increase in pulmonary vascular resistance and a decrease in the 6-min walk test (3, 4). The consequences of perivascular inflammation include cytokine production by vascular and inflammatory cells, and degradation of the extracellular matrix (ECM) by proteases (5, 6). Both the increased cytokine production and the peptides that are released as a result of ECM degradation cause activation and recruitment of circulating immune cells (6, 7). Neutrophils are among the cells that are recruited, and these cells release proteolytic enzymes, including neutrophil elastase (NE), that cause vascular injury (8). Although many aspects of perivascular inflammation can cause progressive PAH, this review will focus on neutrophils and NE. We will review evidence of neutrophil accumulation in PAH, discuss the role of NE and other proteases in driving vascular remodeling and highlight potential interactions between neutrophils, NE and the key genetic driver of PAH, bone morphogenetic protein receptor type 2 (BMPR2).

## Neutrophils and PAH

Neutrophils are the predominant circulating leukocyte population and are important in modulating innate and adaptive immunity. They are early responders and are recruited to sites of sterile inflammation and infection by environmental cues.

Relatively little attention has been given to the role of neutrophils in the pathogenesis of PAH. Neutrophil and macrophage perivascular accumulation has been observed in murine lungs in association with hypoxic pulmonary hypertension (PH) and in monocrotaline-induced PH in rats (9, 10). It has also been established that the neutrophil to lymphocyte ratio is increased in PAH patients when compared to healthy controls (11) and that increased neutrophil to lymphocyte ratio positively correlates with New York Heart Association (NYHA) functional class (FC) and predicts event free survival (12, 13). It is not clear why this relative increase in circulating neutrophils is associated with PAH progression. However, neutrophils produce a wide range of substances that could contribute to vascular remodeling and promote inflammation in PAH. For example, myeloperoxidase (MPO), a catalyst for reactive oxygen species (ROS) formation, has recently been implicated in the pathophysiology of PAH (14). Two independent cohorts of PAH patients had increased plasma MPO relative to healthy controls and *Mpo*^−/−^ mice displayed reduced right ventricular pressures following hypoxic exposure (14). Interestingly, although MPO can reduce nitric oxide (NO) bioavailability in other vascular beds (15), there was no evidence supporting a difference in NO availability between wild-type and MPO-deficient mice (14). Instead, activation of a Rho kinase pathway by MPO was found to drive pulmonary vasoconstriction and SMC proliferation (14).

In addition to MPO and ROS, neutrophils produce proteolytic enzymes. Activity of these enzymes is tightly controlled by endogenous inhibitors, but excessive protease activity has the potential to destroy tissue and cause extensive fibrotic remodeling, leading to organ failure (16). Indeed neutrophil proteases are implicated in airway and parenchymal lung diseases [reviewed by Taggart et al. (17)] and are known to have roles in systemic vascular pathology (18, 19).

Neutrophil granule proteases include members of the serine protease family (NE, cathepsin G, proteinase 3) and matrix metalloproteinases [neutrophil collagenase (MMP-8) and gelatinase (MMP-9)] (20). Of these proteases, NE has been most heavily implicated in the pathogenesis of PAH, as described below.

## Sources of neutrophil elastase in PAH

Neutrophils are the dominant cellular source of NE but it is also produced by macrophages and smooth muscle cells (SMC) (21–24). There is evidence of both augmented NE release from neutrophils isolated from PAH patients (25) and upregulation of endogenous NE in PAH patient SMCs and in experimental animal models of PH (23, 26, 27). Macrophages are observed in the plexiform lesions of PAH patient lungs and activated macrophages release leukotriene B4, which induces endothelial cell (EC) injury and results in EC apoptosis, but could also promote neutrophil recruitment (28). Moreover, *in vitro* work suggests that human alveolar macrophages internalize NE through ingestion of apoptotic neutrophils, and act as a vehicle for the enzyme, transporting it to the tissues and subsequently releasing the active form (29). However, further work is required to show conservation of this mechanism in perivascular or interstitial lung macrophages.

NE is dispensed by neutrophils during degranulation and upon release of neutrophil extracellular traps (NETs). NETs consist of decondensed chromatin decorated with NE and other antimicrobial proteases. NE plays an important role in NET release, evidenced by reduced NET formation in the presence of NE inhibitors and in NE knockout mice (30). Upon stimulation, NE is discharged from azurophilic granules and translocates to the nucleus where it contributes to histone degradation, facilitating chromatin decondensation (30). Depending on the stimulus, chromatin is then expelled via vesicles (31) or suicidal NETosis (32), reviewed by Jorch et al. (33).

NETs assist in microbial trapping and killing (32), but non-infectious roles for NETs are emerging and NETs have been identified in vascular pathologies such as atherosclerosis and PAH (34, 35). Detrimental consequences attributed to NETs include nuclear factor kappa-light-chain-enhancer of activated B cells-dependent PA EC pathological angiogenesis, release of the vasoactive agent endothelin-1, and promotion of SMC proliferation (34). NETs can also directly induce EC cell death (36) and promote thrombus formation (37). In PAH lung tissue, markers of NET formation (DNA, myeloperoxidase, and citrullinated histone H3) have been identified in proximity to plexiform lesions and circulating DNA levels were elevated in IPAH patient plasma (34), although this study did not investigate whether IPAH patient neutrophils are more prone to releasing NETs.

Studies prior to the identification of NETs had suggested that binding of NE to DNA inhibited the activity of the protease (38). However, Kolaczkowska et al. used an *in vivo* zymography assay to demonstrate that NE associated with NETs remains proteolytically active (39). This implies that NE attached to NETs is shielded from endogenous anti-protease activity and thus high NET levels in PAH may represent an important source of active NE.

## Neutrophil elastase in the pathology of PAH

Elastolytic activity has been implicated in the pathogenesis of PAH for over three decades, with evidence of fragmented internal elastic laminae in the pulmonary arteries of children with congenital heart defects (40). There is a clear temporal relationship between increased NE activity and vascular changes in PAH experimental rat models (41). Furthermore, inhibition of NE enzymatic activity not only attenuates the progression of PAH but can also reverse the disease process in experimental models. This was first shown in the monocrotaline model where a progressive and fatal form of PH was reversed by elastase inhibitors in association with normalization of hemodynamic changes of PH and structural abnormalities that included extensive occlusive muscularization (26). Nickel et al. also demonstrated reversal of hemodynamic and histological measures of PH in the Sugen/Hypoxia rat model following treatment with recombinant human elafin (42). Increased NE activity was demonstrated in the lungs of rats with Sugen/Hypoxia-induced PH and this was attenuated by elafin (42). Importantly, relevance to human disease was confirmed through use of a human pulmonary artery explant model. Tissue sections containing pulmonary arteries were isolated from the explanted lungs of PAH patients and treatment with elafin induced regression of neointimal changes and improved measures of vessel lumen size (42).

Release of NE can stimulate adverse remodeling by degrading virtually all the components of the ECM in addition to elastin, and including collagen, fibronectin, and laminin (43). As a consequence, degraded ECM releases bioactive peptides and growth factors such as epidermal growth factor (EGF) and fibroblast growth factor (FGF), which have both mitogenic and motogenic effects on SMCs and fibroblasts (44–47). Additionally, heightened NE activity leads to the activation of matrix metalloproteinases (MMPs), which could potentiate ECM degradation, and induction of tenascin C, a glycoprotein associated with the upregulation of growth factor receptors and proliferation of SMCs (48). The sub-endothelial deposition of tenascin C and fibronectin appears to be a chemotactic factor for PA SMCs, facilitating their migration and the formation of neointimal lesions (48, 49). Conversely, inhibition of NE leads to reduced tenascin C, induction of SMC apoptosis, and regression of pulmonary artery (PA) medial hypertrophy (26).

The role of elastase in vascular remodeling was further explored using transgenic mice that over-express the S100A4 protein (23, 27). Following infection with the murine herpes virus, MHV-68, S100A4 mice develop histological features of human PAH including neointima formation (27). These vascular lesions display fragmented elastic laminae associated with heightened lung elastase activity (27). Infusion of recombinant human elafin, an endogenous elastase inhibitor, reduced the number and severity of neointimal lesions in this model (23). Furthermore, using FLAG-tagged elafin, NE was identified as the serine elastase responsible for the elevation in elastase activity in the S100A4 lungs (23). While this finding implies that NE is the dominant target in this model, the relative importance of NE above other proteases in human disease has not been confirmed and the potential roles of other proteases will be discussed below.

The source of NE in the S100A4 lungs was localized not only to neutrophils but also to PA SMCs and, as indicated above, this finding was conserved in human disease, evidenced by cultured PA SMCs from IPAH patients expressing higher levels of NE than cells from control donor lungs (23). Interestingly, *ex vivo* perfusion of S100A4 lungs with porcine pancreatic elastase suggested that the elastin fibers in these mice were more prone to degradation (23). This implies that certain viral infections, and potentially other inflammatory stimuli, may predispose the vasculature to pathological remodeling upon exposure to elastase.

## Neutrophil elastase and perpetuation of inflammation in PAH

In addition to the impact of NE on SMC migration and proliferation, this enzyme contributes to PAH pathogenesis by proteolytic modification of cytokines. For example, NE promotes IL-1β activity by cleaving the pro-isoform of IL-1β in human coronary ECs, thereby increasing the secretion of the bioactive form in extracellular vesicles (50). Notably, secretion of active IL-1β could promote neutrophil survival, an example of positive regulation of neutrophil activity by cytokines (51).

NE also cleaves CXCL12 (SDF-1 alpha), a chemokine involved in the regulated release of neutrophils from the bone marrow (52–54). CXCL12 inactivation would promote mobilization of neutrophils into the circulation, particularly in the context of elevated plasma IL-8 levels, as observed in PAH patients (4, 54). On the other hand, NE can inactivate tumor-necrosis factor (55) and IL-6 (56), suggesting that there is a balance that must be maintained between elastase and anti-elastase activity. The chronic perivascular inflammatory phenotype of PAH patients characterized by elevated levels of circulating and tissue cytokines, is consistent with persistent neutrophil activation. Indeed, many of the cytokines elevated in PAH are known to enhance neutrophil function and survival and may thus perpetuate neutrophil-mediated inflammation (Table 1).

**Table 1:** Cytokines elevated in PAH and known to alter neutrophil function.

**Cytokine**	**Release**	**Priming**	**Adhesion**	**Chemotaxis**	**ROS**	**Survival**	**References**
CCL2	**x**		**x**	**x**		**x**	(57)
IL-1α	**x**						(58)
IL-1β	**x**	**x**	**x**	**x**	**x**	**x**	(4, 58, 59)
IL-4	**x**						(4)
IL-6	**x**		**x**	**x**			(4, 58–60)
IL-8	**x**	**x**	**x**	**x**			(4)
IL-10	**x**						(4)
IL-12	**x**						(4)
Interferon- γ	**x**	**x**				**x**	(4)
TNF-α	**x**	**x**	**x**	**x**	**x**	**x**	(4, 58)

*The x symbols indicate which functions are altered by each cytokine. ROS, reactive oxygen species generation; CCL2, C-C Motif Chemokine Ligand 2, also known as monocyte chemoattractant protein 1; IL, interleukin; TNF, tumor necrosis factor*.

## Other proteases implicated in tissue remodeling in PAH

As mentioned above, other neutrophil proteases may contribute to the vascular remodeling observed in PAH. For example, MMP-9 expression is upregulated in human plexiform pulmonary arterial lesions (61) and in lungs isolated from rats with monocrotaline-induced pulmonary hypertension (62). Furthermore, transgenic overexpression of human MMP-9 exacerbated monocrotaline-induced pulmonary hypertension in mice (63). The role of other MMPs and MMP inhibitors in PAH has been reviewed by Chelladuri et al. (64).

This review has focused mainly on neutrophil proteases, but it should be noted that many other cells release proteases. For example, upregulation of MMP-9 has been detected in natural killer cells from patients with PAH (65) and it is also released by monocytes and macrophages (66). Further examples of important non-neutrophil proteases are chymase and tryptase, the main proteases released by mast cells. Heath and Yacoub (67) noted increased perivascular mast cell numbers in both primary and secondary forms of pulmonary hypertension compared to controls, an observation confirmed more recently by others (68, 69). Circulating tryptase levels were elevated in PAH patients compared to controls and correlated with disease severity as assessed by brain natriuretic peptide level (68). In this study a small number of patients were treated with mast cell stabilizers, but there were no changes in clinical endpoints such as 6-min walk distance or BNP level.

A key question about protease activity in any disease process is how the protease evades suppression by endogenous inhibitors. NE is inhibited by several anti-proteases including α1-antitrypsin, secretory leucocyte peptidase inhibitor (SLPI) and elafin. On the one hand it is possible that localized release of the protease simply overcomes anti-protease activity in the immediate microenvironment. Indeed, markers of pre-inhibited elastase activity have been reported including the cleaved fibrinogen product, Aα-Val^360^ (70). On the other hand, proteases may be shielded from their inhibitors by attachment to NETS (discussed above), or via transport in exosomes. Neutrophil-derived exosomes degrade NE substrates and induce emphysematous changes in murine lungs following intra-tracheal administration (71). It is also possible that proteases have enzyme-independent functions but the beneficial consequences of elafin treatment in animal models of PH and in lung explant models (23, 42) would favor the hypothesis that there is an excess of protease activity. Interestingly, however, elafin may exert protective effects independently of protease inhibition as described below.

## Neutrophils and PAH related to BMPR2 deficiency

The bone morphogenetic protein receptor 2 (BMPR2) signaling pathway has become a key focus of investigation since *BMPR2* gene mutations were identified as the main predisposing risk factor in the heritable forms of PAH (HPAH) (72), with dysfunction in the signaling pathway present in all subtypes of PAH (73). However, although present in a high proportion of HPAH cases—identified in 70% of familial PAH cases (74)—the autosomal dominant *BMPR2* mutation exhibits low penetrance with 70–80% of those carrying the mutation never developing PAH (75). This, and the lack of spontaneous PAH in most heterozygous *BMPR2* animal models, implies that a second insult or background genetic variants are needed for predisposed individuals to develop PAH. Inflammation has been proposed as a second hit which promotes adverse remodeling when there is a loss of BMPR2.

BMPR2 is expressed by all cells in the arterial wall but by far the highest level of expression is present in endothelial cells (73). Reduced BMPR2 signaling is related both to excessive vascular smooth muscle proliferation (76) and exaggerated PA EC apoptosis (77). A study by Burton et al. (78) investigated the role of BMPR2 in maintaining the barrier function of PA ECs and in suppressing inflammation within the pulmonary vasculature. Using static and flow-based *in vitro* systems, they were able to demonstrate that a reduction in BMPR2 expression facilitated neutrophil transmigration across the PA EC monolayer and reported that a lack of BMPR2 led to overexpression of IL-8, which in turn led to the recruitment of neutrophils. Similarly, loss of BMPR2 can lead to heightened expression of IL-6, an inducer of SMC proliferation (79). Taken together, BMPR2 plays a role in dampening inflammatory signals in the pulmonary vasculature that could influence neutrophil recruitment and elastase activity.

Conversely, neutrophils may also impact BMPR2 function by releasing NE and degrading BMP9, an anti-angiogenic ligand for the BMPR2/ALK1 heterodimer present on PA ECs (80). BMP9 has been shown to circulate in humans at biologically active levels (81), maintaining vascular quiescence. Li et al. showed that BMP9 is readily cleaved by NE, released by activated neutrophils (80). Further work by this group demonstrated that the administration of recombinant BMP9 reversed established PH in *BMPR2*-deficient mice, overcoming reduced BMPR2 levels, and preventing lung vascular leakage (82). Paradoxically, Appleby et al. demonstrated that BMP9 could have a pro-inflammatory role and, in fact, facilitate neutrophil recruitment to the pulmonary vasculature by activated endothelial cells (83). However, the mouse model used in that study was acute endotoxemia, so this may not relate directly to PAH pathogenesis.

Overall, it appears that inflammation-driven release of NE can suppress BMPR2 signaling. Nickel and colleagues investigated the impact of the NE inhibitor elafin on BMPR2 signaling in PAH (42). In the Sugen/Hypoxia rat model of PH, elafin improved pulmonary endothelial expression of apelin, a BMPR2 target gene. Loss of apelin expression in the pulmonary endothelium during PAH is associated with the failure to repress the release of fibroblast growth factor-2 (FGF2) (84). Nickel et al. also found that in human cells, elafin augmented BMPR2 interactions with caveolin-1, another downstream target of BMPR2 signaling (42). Unfortunately, changes in BMP9 levels following elafin administration were not measured. Nonetheless, the findings suggest that improving BMPR2 signaling in addition to the beneficial sequelae of inhibiting NE, described earlier in this review, could reverse the vascular remodeling observed in PAH. Interestingly, targeting NE activity may be of particular importance in patients with BMPR2 mutations, as histological assessment of pulmonary vessels in such patients demonstrates a reduction in elastin and fibrillin-1, the two major constituents of elastic fibers (85). Furthermore, pulmonary artery elastic fibers from mice with compound *Bmpr2/1a* mutations were more susceptible to elastase-mediated degradation of elastic fibers compared to wild-type mice (85).

## Summary

**Figure 1:**
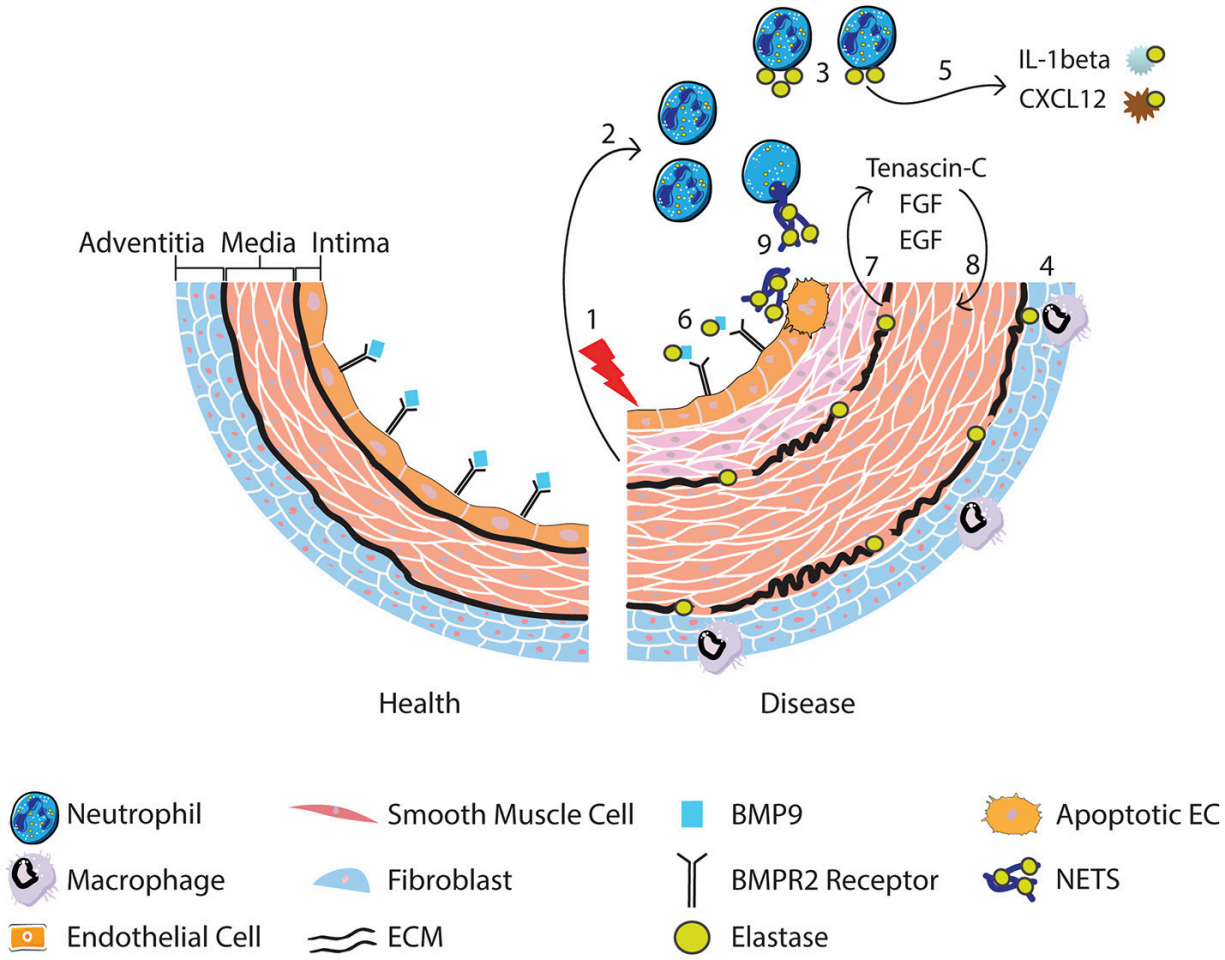
Following vascular injury (1), the release of chemokines and chemotactic peptides from surrounding tissue recruits and activates neutrophils (2). Neutrophils release elastase (3) although it can also be released from activated macrophages that engulf neutrophil elastase (4) and from smooth muscle cells. Release of neutrophil elastase leads to cleavage of cytokines resulting in the conversion of the pro-to active form of IL-1b, promoting neutrophil survival, and degradation of CXCL12, favoring release of neutrophils from the bone marrow (5). BMP9 is also cleaved, impacting upon BMPR2 receptor signaling (6). Degradation of the ECM by elastase (7) releases SMC mitogenic growth factors promoting SMC proliferation (8). Neutrophil elastase is also involved in the release of NETs, which may induce EC apoptosis (9) and therefore contribute to endothelial dysfunction.

Collectively the studies discussed above implicate the neutrophil and neutrophil products such as MPO, proteases and NETs in the pathogenesis of PAH. Neutrophils are attractive candidates in contributing to vascular changes seen in PAH because they are among the first cells to arrive at sites of inflammation. The evidence of increased circulating elastase in PAH patients and fragmented elastic lamina in PAH vessels, highlights a key pathogenic role for proteolytic enzymes and in particular NE. NE has a wide range of targets, but we describe evidence for how it could contribute to PAH pathogenesis by modulating the activity of cytokines and degrading ECM releasing growth factors that promote remodeling. Moreover, the release of NE and NETs is likely to alter the local inflammatory environment, augmenting leukocyte responses and further driving inflammation in PAH. It is also evident that reduced BMPR2 receptor signaling cooperatively interacts with the sequelae of inflammation and neutrophil activation in contributing to adverse vascular remodeling. Figure 1 provides a summary of the role of neutrophils, NE and NETs in PAH.

While increases in NE activity coincide with changes in vascular remodeling in animal models, it remains unclear whether intrinsic neutrophil abnormalities are necessary to initiate aberrant remodeling in human lungs or whether alterations in NET and NE release by neutrophils are a consequence of other features of the disease. To better understand the role of neutrophils at different stages of PAH pathogenesis, a longitudinal study to evaluate how they become increased and activated would be important. However, directly targeting neutrophils as a therapeutic strategy would be challenging given their vital role in host defense and as mentioned above, cells other than neutrophils can produce damaging proteases. Of these proteases, NE provides an attractive target as evidence from animal models suggests that NE inhibition has the potential to inhibit aberrant remodeling of the pulmonary vessels and indirectly dampen persistent inflammation in PAH. These findings have encouraged initiation of clinical trials of elastase inhibitors such as elafin for PAH (NCT03522935). Further, the potential for NE inhibitors such as elafin to enhance BMPR2 signaling and target pathology driven by BMPR2 deficiency, identifies these drugs as promising novel therapies for PAH.

## Author contributions

ST and OD wrote sections of the manuscript; AT initiated the work and drafted sections of the manuscript; AT, MR and RZ revised the manuscript critically for important intellectual content.

### Conflict of interest statement

AT has received funds to attend educational events from Actelion. RZ has a patent for use of FK506 to treat pulmonary hypertension. RZ has performed consultancy work for Actelion and Vivus and has stock options with Selten. The remaining authors declare that the research was conducted in the absence of any commercial or financial relationships that could be construed as a potential conflict of interest. The reviewer JS and handling Editor declared their shared affiliation.
